# A Scene-Aware Confidence-Guided YOLO Framework for Robust Weld Seam Tracking Under Intense Arc Interference

**DOI:** 10.3390/s26154861

**Published:** 2026-08-02

**Authors:** Lin Gao, Feichi Cai, Xiaobo Shi

**Affiliations:** 1 School of Electrical Engineering, Yanshan University, Qinhuangdao 066004, China; shixiaobo@hbjcxy.edu.cn; 2 The 54th Research Institute of China Electronics Technology Group Corporation, Shijiazhuang 050050, China; 3 International School of Transportation, Shijiazhuang Institute of Railway Technology, Shijiazhuang 050062, China; tianshi20066@163.com; 4 Department of Information Engineering, Hebei Construction Material Vocational and Technical College, Qinhuangdao 066004, China

**Keywords:** weld seam tracking, scene-aware confidence, YOLO, measurement reliability, Kalman filtering

## Abstract

Accurate weld seam tracking under severe arc interference remains a major challenge for intelligent robotic welding systems because radiometric saturation and transient visual degradation significantly reduce the reliability of vision-based measurements. Existing YOLO-based tracking methods generally use detector outputs directly for state estimation, assuming that the detection confidence adequately reflects localization reliability. However, this assumption often breaks down under strong arc interference, leading to unstable tracking and occasional tracking failure. To address this problem, this paper proposes a scene-aware confidence-guided YOLO (SACG-YOLO) framework for robust weld seam tracking. The proposed method introduces a scene-aware confidence estimator that evaluates the observability of the current welding scene by jointly considering structural consistency and photometric variation. The estimated scene confidence is fused with the detector confidence to assess measurement reliability, which is subsequently used to adaptively regulate the Kalman filter update process. As a result, reliable observations are incorporated into state estimation, while unreliable measurements are rejected in favor of motion prediction, thereby improving tracking continuity during severe visual degradation. Experiments on industrial welding image sequences demonstrate that the proposed framework consistently outperforms conventional YOLO-based tracking methods. Under stable and weak arc interference, the average tracking error is reduced from 0.492 mm to 0.155 mm, while the maximum error decreases from 0.829 mm to 0.354 mm. Under severe arc interference, where standalone YOLO tracking fails, SACG-YOLO maintains continuous weld seam localization with a maximum tracking error of 0.406 mm. These results demonstrate that explicitly modeling measurement reliability through scene-aware confidence estimation significantly improves the accuracy, robustness, and continuity of weld seam tracking in complex industrial welding environments.

## 1. Introduction

Welded hollow spheres serve as critical structural connectors in spatial grid steel structures and are widely used in large-span engineering applications, such as industrial plants, gymnasiums, and airport terminals [1]. Weld seam tracking is a fundamental task in automated welding systems, directly affecting welding quality and efficiency. However, due to the diversity of sphere sizes and complex geometries, most existing welding robots still rely on the “teach-playback” mode, which significantly limits their flexibility, adaptability, and level of automation.

With the rapid advancement of machine vision and intelligent sensing technologies, structured-light vision–based weld seam detection and tracking have emerged as effective solutions for intelligent welding. A typical system consists of a line laser, a vision sensor, and an industrial computer. The laser projects a stripe onto the weld surface to enhance geometric features, while the vision sensor captures the stripe image, often using optical filters to suppress arc interference. The acquired images are then processed to extract weld features and guide real-time torch motion [2,3,4].

Traditional image-processing methods have been widely applied to weld seam detection and tracking. For example, contour scanning [5], filtering-based feature extraction [6], and model-based approaches such as NURBS-snake models [7] have been used to locate weld features. In addition, tracking methods based on optical flow, particle filtering, and Bayesian similarity have been proposed to improve robustness [8,9,10,11]. Kalman filtering has also been introduced to enhance temporal consistency in structured-light systems [12]. While these methods are computationally efficient and interpretable, their performance degrades under complex conditions such as dynamic deformation, low contrast, and noise.

In recent years, deep learning-based methods have significantly advanced weld seam detection and tracking. Representative studies have employed Faster R-CNN, Mask R-CNN, and various YOLO architectures to improve weld seam localization accuracy and robustness [13,14,15,16,17,18,19,20,21] For example, Liu et al. proposed an improved YOLOv8s-Seg framework for weld seam tracking and inspection robots, achieving enhanced detection accuracy and real-time performance through joint segmentation and localization [20]. Similarly, WeldNet introduced a dedicated deep neural architecture for weld seam type identification and initial point guidance, demonstrating the effectiveness of deep learning for intelligent welding applications [22]. In addition, keypoint-based deep learning methods have been proposed to improve seam localization accuracy and facilitate trajectory tracking in robotic welding systems [23]. These studies demonstrate the strong capability of deep learning models for weld seam feature extraction and semantic localization.

To improve perception performance under challenging welding conditions, recent research has increasingly focused on mitigating the effects of arc glare, smoke, spatter, and illumination variations. For instance, Yang et al. proposed a Local-Add U-Net (LAU-Net) to enhance laser stripe extraction under strong interference in laser–arc hybrid welding environments [24]. Other studies have combined two-dimensional images with three-dimensional point clouds to improve weld seam detection robustness in visually degraded scenarios [25]. Furthermore, recent reviews on robotic welding vision systems have highlighted the growing importance of robust visual sensing and perception reliability for intelligent welding applications [26]. Nevertheless, most existing studies primarily focus on improving feature extraction capability, network architecture design, or detection accuracy under interference, while comparatively little attention has been paid to the reliability of detector outputs during tracking.

Meanwhile, reliability estimation and uncertainty quantification have emerged as active research topics in the broader computer vision community. Recent studies have shown that the confidence scores reported by modern object detectors are often poorly calibrated and may not accurately reflect prediction reliability, particularly under distribution shifts, occlusions, and visually degraded environments [27,28]. To address this issue, confidence-consistent learning frameworks and uncertainty-aware object detectors have been proposed to improve the consistency between confidence estimation and localization quality [29,30]. Furthermore, recent investigations on detector calibration have demonstrated that high-confidence predictions do not necessarily correspond to high localization accuracy, revealing a potential mismatch between detector confidence and actual prediction reliability [31].

Despite these advances, the problem remains largely unexplored in robotic weld seam tracking under severe arc interference. Existing welding-oriented tracking methods generally assume that detector outputs can be directly used as reliable measurements for subsequent state estimation, and detector confidence is therefore implicitly regarded as an indicator of observation reliability [20,21,22,23,24]. However, this assumption does not always hold in practical welding environments. Under severe arc glare, metal spatter, and radiometric saturation, the visual observability of the weld seam can deteriorate substantially, leading to transient feature loss, structural distortion, and unstable image formation. As the scene degradation intensifies, YOLO may either fail to detect the weld seam or still produce detections with relatively high confidence despite substantial localization errors. Consequently, detector confidence and localization reliability become progressively decoupled, allowing unreliable observations to be incorporated into the tracking process and resulting in state contamination, error accumulation, trajectory drift, and even tracking failure. Therefore, robust weld seam tracking requires not only accurate object detection but also explicit modeling of scene degradation and observation reliability before visual measurements are incorporated into state estimation.

To address this limitation, this paper proposes a scene-aware confidence-guided YOLO (SACG-YOLO) framework for robust and continuous weld seam tracking under severe arc interference. Instead of directly using detector outputs for state updating, the proposed framework first evaluates the degradation level of the current welding scene within a dynamically updated weld Region of Interest (ROI) by jointly analyzing structural consistency and photometric variation. The resulting scene-aware confidence explicitly characterizes weld seam observability under varying arc interference conditions and is further fused with YOLO confidence to estimate the reliability of detection results. Rather than updating the Kalman filter with every detector output, the proposed framework performs reliability-aware adaptive measurement regulation, whereby only reliable observations are incorporated into state estimation while degraded measurements are automatically rejected. By integrating scene-aware reliability evaluation, confidence-guided measurement regulation, Kalman filter-based temporal prediction, and closed-loop ROI feedback, the proposed framework transforms weld seam tracking from conventional detector-driven measurement updating into a reliability-aware state estimation process capable of adapting to dynamically changing observation conditions.

The main contributions of this work are summarized as follows:

(1) A scene-aware reliability modeling mechanism is proposed to explicitly characterize weld seam observability under varying arc interference conditions. By jointly evaluating structural consistency and photometric variation within the weld Region of Interest (ROI), the proposed method quantitatively estimates scene degradation and provides a physically interpretable scene-aware confidence for reliability assessment prior to state estimation.
(2) A reliability-aware adaptive measurement regulation strategy is developed by jointly considering scene-aware confidence, YOLO confidence, and motion-consistency constraints. The proposed strategy selectively incorporates reliable observations into the Kalman state estimation process while rejecting degraded measurements, thereby preventing state contamination, suppressing error propagation, and improving tracking robustness under severe visual degradation.
(3) A closed-loop reliability-aware weld seam tracking framework, termed SACG-YOLO, is established by tightly coupling YOLO-based semantic detection, Kalman filter prediction, scene-aware reliability evaluation, and ROI feedback localization. Unlike conventional detector-driven tracking methods, the proposed framework maintains spatial-temporal tracking continuity even during temporary detector failures or severe arc-induced visual degradation, thereby providing a lightweight, robust, and practically deployable solution for intelligent robotic weld seam tracking in complex industrial environments.

The remainder of this paper is organized as follows. Section 2 introduces the proposed SACG-YOLO framework, including the scene-aware reliability modeling mechanism, confidence-guided adaptive measurement regulation strategy, Kalman filter-based temporal prediction, and the closed-loop tracking framework. Section 3 presents the experimental setup and comprehensively evaluates the proposed method through training analysis, comparative tracking experiments under nominal, weak arc interference, and severe arc interference conditions, together with reliability analysis and ablation studies. Section 4 discusses the underlying mechanisms of the proposed reliability-aware tracking framework, analyzes its robustness under dynamically varying observation conditions, and further examines its practical implications, limitations, and future extensions. Finally, Section 5 concludes the paper and outlines directions for future research.

## 2. Materials and Methods

### 2.1. Framework Overview

**Figure 1:**
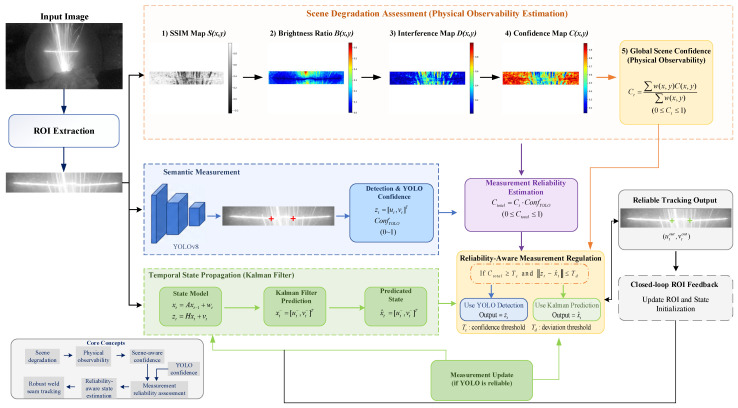
Overview of the proposed SACG-YOLO framework. Scene degradation is first assessed to estimate physical observability, which is subsequently fused with YOLO confidence to estimate measurement reliability for reliability-aware state estimation and closed-loop weld seam tracking under severe arc interference.

The overall architecture of the proposed SACG-YOLO framework is illustrated in Figure 1. The framework is designed to achieve robust and continuous weld seam tracking under severe arc interference by explicitly incorporating scene degradation assessment into the measurement update process. Unlike conventional YOLO-based tracking methods that directly use detector outputs as measurement observations, the proposed framework evaluates the degradation level of the current welding scene and estimates the reliability of each detection before it is incorporated into state estimation. This reliability-aware design enables the tracking system to adaptively regulate measurement utilization under dynamically varying welding conditions.

The proposed framework consists of three major functional modules: a YOLOv8-based semantic measurement module, a scene-aware confidence evaluation module, and a Kalman filter-based temporal prediction module. These modules are tightly integrated through a confidence-guided adaptive measurement regulation mechanism to maintain robust spatial-temporal tracking continuity under severe arc interference.

Given an input welding image, a Region of Interest (ROI) containing the weld seam is first extracted to reduce computational complexity and suppress irrelevant background interference. Within the extracted ROI, three processing branches are executed in parallel.

The first branch is the semantic measurement module, where YOLOv8 performs weld seam detection and outputs a candidate weld position together with the corresponding detector confidence score. Under normal and weak arc interference conditions, YOLO generally provides accurate semantic localization with high confidence.

The second branch is the scene-aware confidence evaluation module. This module explicitly evaluates the degradation level of the current welding scene by jointly analyzing structural consistency and photometric variation within the ROI. By quantifying the influence of arc glare, radiometric saturation, metal spatter, and local structural degradation, the proposed method estimates the observability of the weld seam and generates a scene-aware confidence score. Unlike detector confidence, which reflects the detector’s prediction confidence, the proposed scene-aware confidence provides an independent estimate of observation reliability based on the physical imaging condition. Consequently, it complements detector confidence when determining whether the current detection can be regarded as a reliable measurement for state estimation.

The third branch is the temporal prediction module, which employs a Kalman filter to propagate the weld seam state from the previous frame and generate an a priori prediction of the current weld position. This predicted state preserves temporal continuity when YOLO detections become unreliable or temporarily unavailable due to severe arc interference and visual degradation.

To assess the reliability of the current observation, the scene-aware confidence is fused with the YOLO confidence to construct a joint confidence metric. This metric jointly reflects detector confidence and scene observability, providing a more reliable measure of observation quality than either component alone. Based on the joint confidence together with a motion-consistency constraint, the framework performs reliability-aware adaptive measurement regulation. Only when both constraints are satisfied is the YOLO detection accepted as a valid measurement for Kalman filter updating; otherwise, the unreliable observation is rejected, and the tracking state is propagated using temporal prediction alone. This adaptive regulation mechanism effectively prevents degraded measurements from contaminating the state estimation process under severe arc interference.

Finally, the validated tracking result is fed back to update the ROI location and initialize the tracking state of the subsequent frame, thereby forming a closed-loop tracking architecture. Through the integration of scene degradation assessment, reliability-aware measurement regulation, temporal state propagation, and ROI feedback localization, the proposed SACG-YOLO framework effectively mitigates the mismatch between detector confidence and localization reliability under severe arc interference. Consequently, the proposed framework transforms conventional detector-driven weld seam tracking into a reliability-aware state estimation process, achieving robust and continuous weld seam tracking under dynamically changing observation conditions.

### 2.2. Scene Degradation Assessment and Confidence Modeling

Arc welding environments are frequently affected by severe arc glare, metal spatter, smoke, and molten pool reflections. These disturbances may cause radiometric saturation, structural degradation, and transient feature loss within the weld seam region, substantially degrading the visual observability of the weld seam. Under such conditions, the semantic confidence reported by YOLO no longer necessarily reflects the actual localization reliability of the detected weld seam. In particular, severe arc-induced visual degradation may preserve semantically plausible image patterns while simultaneously introducing significant geometric localization errors. Consequently, detector confidence and localization reliability become progressively decoupled, making detector confidence alone insufficient for reliable measurement selection.

To address this limitation, the proposed framework explicitly models the degradation level of the current welding scene and incorporates this information into the reliability assessment of YOLO detections. Rather than directly treating detector confidence as an indicator of observation quality, the proposed method first evaluates the physical observability of the weld seam within a dynamically updated Region of Interest (ROI). Specifically, a scene-aware confidence modeling strategy is developed by jointly analyzing structural consistency and photometric variation within the ROI. The resulting scene-aware confidence quantitatively characterizes the degree of scene degradation and serves as an independent reliability cue for determining whether the confidence reported by YOLO can be regarded as a trustworthy indicator of localization quality.

The following subsections describe the structural consistency modeling, photometric variation modeling, local confidence estimation, and global confidence aggregation procedures used to construct the proposed scene-aware confidence.

#### 2.2.1. Structural Consistency Modeling

Let $I_{t}(x,y)$ denote the grayscale intensity of the current frame at pixel (x,y), and let $I_{ref}(x,y)$ denote the corresponding pixel intensity in a reference frame within the ROI Ω. To quantify the loss of local structural observability caused by arc-induced visual degradation, the Structural Similarity Index (SSIM) is computed within a local window centered at each pixel:

(1) $$S(x,y)=\frac{\left(2\mu_{t}\mu_{ref}+C_{1})(2\sigma_{t,ref}+C_{2}\right)}{\left(\mu_{t}^{2}+\mu_{ref}^{2}+C_{1})(\sigma_{t}^{2}+\sigma_{ref}^{2}+C_{2}\right)}$$

where $\mu_{t}$ and $\mu_{ref}$ are local mean intensities, $\sigma_{t}^{2}$ and $\sigma_{ref}^{2}$ are local variances, and $\sigma_{t,ref}$ denotes the covariance. Constants $C_{1}$ and $C_{2}$ used to maintain numerical stability. Lower SSIM values indicate reduced structural observability, suggesting that the current visual measurements are less reliable for weld seam localization.

#### 2.2.2. Photometric Variation Modeling

To characterize local photometric degradation caused by arc interference, a brightness ratio is introduced to measure pixel-wise intensity variation between the current frame and the reference frame:

(2) $$R(x,y)\frac{I_{t}(x,y)}{I_{ref}(x,y)+\varepsilon }$$

where ε is a small constant to avoid division by zero. Since severe arc interference primarily manifests as abnormal radiometric saturation and localized overexposure, only positive intensity deviations are retained to construct the photometric variation map.

(3) $$B\left(x,y\right)=max\left(0,R\left(x,y\right)\right)$$

#### 2.2.3. Structure-Modulated Interference Modeling

Because weld seams and arc interference may both exhibit strong intensity responses, structural consistency and photometric variation are jointly integrated to estimate the local degradation level of the welding scene. The interference response map is defined as

(4) D(x,y)=(1−S(x,y))+(α+β⋅B(x,y))

where α and β control the contribution of structural degradation and photometric variation, respectively.

Based on the estimated degradation response, a pixel-wise scene confidence map is subsequently constructed as

(5) $$C(x,y)=\exp(-\frac{D(x,y)}{k})$$

where *k* controls the sensitivity of confidence attenuation. Pixels affected by severe structural degradation or strong photometric disturbance are assigned lower confidence values, indicating reduced local observability and lower measurement reliability.

#### 2.2.4. Global Confidence Estimation

To obtain a global confidence score for the current frame, the pixel-wise scene confidence map is aggregated using a spatial weighting function centered at the predicted weld seam position:

(6) $$C_{t}=\frac{\sum_{(x,y)\in \Omega }w(x,y)C(x,y)}{\sum_{(x,y)\in \Omega }w(x,y)}$$

where w(x,y) is typically implemented as a Gaussian weighting function to suppress background influence and emphasize the weld seam region. The resulting scene-aware confidence lies within the range ([0,1]) and characterizes the overall observability of the weld seam under the current imaging conditions. Higher confidence values indicate that the scene is less affected by arc-induced degradation and that the visual observations are more likely to provide reliable localization information. This scene-aware confidence is subsequently fused with the detector confidence to determine whether the current YOLO observation should participate in the Kalman filter update process.

### 2.3. Measurement Modeling via YOLO Detection

The YOLOv8 detector serves as the semantic measurement module for weld seam localization. Given an input ROI, the detector estimates the weld seam position:

(7) $$z_{t}={\left[u_{t},v_{t}\right]}^{T}$$

together with a detector confidence score:

(8) $$Conf_{YOLO}\in [0,\;1]$$

which reflects the detector’s internal confidence in the predicted localization.

Under nominal welding conditions, YOLO generally provides accurate and reliable weld seam localization. However, under severe arc interference, radiometric saturation, plasma blooming, and transient feature degradation may substantially alter the visual appearance of the weld seam while preserving semantically plausible image patterns. As a result, the detector may still output relatively high confidence scores despite significant localization errors. Therefore, detector confidence alone cannot be regarded as a reliable indicator of measurement quality under severe visual degradation. Although the confidence score reported by YOLO reflects the detector’s internal assessment of prediction quality, severe arc interference may substantially alter the visual characteristics of the weld seam without necessarily causing a proportional decrease in detector confidence. Consequently, high-confidence detections may still exhibit significant localization errors under radiometric saturation and structural degradation. Therefore, the proposed framework does not directly use YOLO confidence as a reliability indicator but instead combines it with scene-aware confidence estimation for subsequent decision-making.

Within the proposed framework, the YOLO output is treated as a candidate measurement observation rather than an automatically trusted measurement. Specifically, the detector output is formulated as

(9) $$z_{t}=Hx_{t}+v_{t}$$

where *H* is the observation matrix and $v_{t}$ denotes measurement noise. Unlike conventional YOLO-based tracking methods that directly utilize detector outputs for state updating, the proposed framework explicitly evaluates the reliability of each measurement by combining detector confidence with scene-aware confidence before it participates in temporal state estimation.

### 2.4. Temporal State Estimation via Kalman Filtering

To maintain temporal continuity under dynamically varying observation reliability, a Kalman filter is employed to model the weld seam motion. The state transition model is expressed as

(10) $$x_{t}=Ax_{t-1}+w_{t}$$

where $x_{t}$ the system state, *A* is the state transition matrix, and $w_{t}$ denotes process noise.

The prediction step generates an a priori estimate of the weld seam position based on previously validated motion states. This predicted state provides stable temporal propagation when visual observations become unreliable due to severe arc interference, temporary feature disappearance, or radiometric saturation.

Unlike conventional Kalman filtering frameworks that update the state whenever detector outputs are available, the proposed framework performs state correction only when the estimated measurement reliability satisfies predefined confidence constraints. Consequently, unreliable observations are prevented from contaminating the internal motion state, while prediction-dominant propagation preserves tracking continuity during periods of severe visual degradation.

### 2.5. Joint Confidence Formulation

Since detector confidence and localization reliability may become inconsistent under severe arc interference, detector confidence alone is insufficient for evaluating measurement quality. To address this issue, the proposed framework introduces a joint confidence formulation by integrating detector-level confidence $Conf_{YOLO}$ and scene-aware confidence $C_{t}$. The joint confidence is defined as

(11) $$C_{total}=C_{t}\cdot Conf_{YOLO}$$

The detector confidence reflects the detector’s internal confidence in its prediction, whereas the scene-aware confidence characterizes the physical observability of the weld seam under the current imaging conditions. These two confidence measures provide complementary information from semantic and physical perspectives.

By jointly considering detector confidence and scene observability, the proposed joint confidence provides a more reliable estimate of measurement quality than either component alone. In particular, when severe arc interference causes detector confidence to become decoupled from localization reliability, the scene-aware confidence effectively suppresses overconfident but unreliable detections, thereby preventing incorrect measurements from entering the temporal estimation process.

### 2.6. Confidence-Guided Decision Strategy

Based on the estimated joint confidence and temporal prediction, an adaptive decision strategy is introduced to regulate the measurement update process. Specifically, the observation is accepted only when it satisfies both a confidence constraint and a deviation constraint relative to the predicted state. Otherwise, the system relies on the prediction alone. The decision rule can be defined as

(12) $$x_{t}^{out}=\left\{\begin{array}{c} z_{t},if\;C_{total}\geq T_{c}\;\mathrm{and}\;\left\|z_{t}-{\hat{x}}_{t}^{-}\right\|\leq T_{d}\;\\ {\hat{x}}_{t}^{-},otherwise \end{array}\right.$$

where $T_{c}$ is confidence threshold, $T_{d}$ denotes deviation threshold.

This dual-constraint strategy ensures that accepted measurements are both physically reliable and temporally consistent. Under nominal conditions, the scene-aware confidence remains consistently high, allowing the framework to fully exploit the localization accuracy of YOLO detections. Under weak arc interference, localized scene degradation leads to moderate reductions in scene confidence, enabling unreliable measurements to be selectively suppressed while retaining reliable detections for state updating. During severe arc interference, scene confidence decreases significantly before detector confidence becomes unreliable, allowing corrupted measurements to be rejected before they contaminate the Kalman filter.

Consequently, the proposed strategy effectively prevents high-confidence but poorly localized detections from degrading temporal state estimation, thereby maintaining stable tracking under dynamically changing interference conditions.

### 2.7. Closed-Loop Feedback Mechanism

To maintain long-term temporal consistency, the proposed framework is formulated as a closed-loop tracking system. The final output $x_{t}^{out}$ is recursively incorporated into the Kalman filtering process, enabling continuous refinement of the state estimate over time.

(13) $${\hat{x}}_{t}=\left\{\begin{array}{c} {\hat{x}}_{t}^{-}+K_{t}(x_{t}^{out}-H{\hat{x}}_{t}^{-}),if\;YOLO\;\mathrm{is}\;\mathrm{selected}\\ {\hat{x}}_{t}^{-},otherwise \end{array}\right.$$

where $x_{t}^{out}$ denotes the decision output. This formulation naturally enables selective updating: when the YOLO measurement is accepted, $x_{t}^{out}=z_{t}$; otherwise, $x_{t}^{out}={\hat{x}}_{t}^{-}$, resulting in no update.

The validated tracking result is simultaneously fed back to dynamically recenter the ROI for the subsequent frame. This feedback mechanism constrains the search region around the predicted weld seam position, suppresses irrelevant background interference, and reduces computational complexity.

More importantly, the closed-loop architecture enables the framework to adapt continuously to dynamically changing observation reliability. During severe visual degradation, prediction-dominant propagation preserves temporal continuity. Once scene observability improves and reliable YOLO measurements become available again, the framework immediately resumes measurement updating without requiring explicit tracker reinitialization. Through the tight integration of scene-aware confidence evaluation, adaptive measurement regulation, temporal prediction, and ROI feedback, the proposed SACG-YOLO framework achieves robust and continuous weld seam tracking under severe arc interference.

## 3. Results

**Figure 2:**
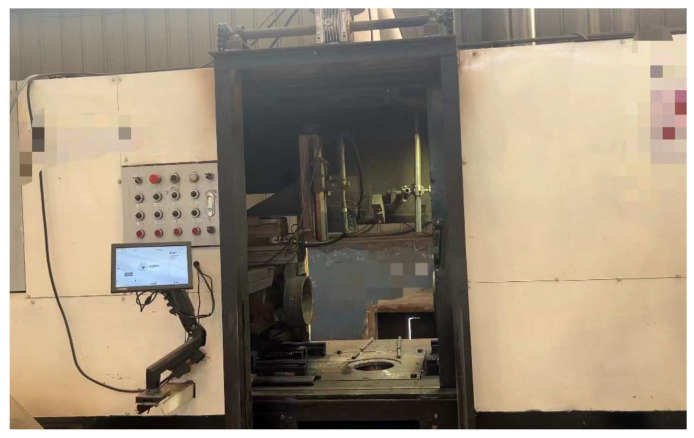
Experimental setup: hollow sphere welding device.

The weld seam image sequences used in this study were acquired using the hollow sphere welding platform shown in Figure 2.

The image acquisition system consists of an industrial camera and a line laser module. The line laser projects a laser stripe onto the weld seam surface to enhance weld geometry and improve feature visibility during welding. To comprehensively evaluate the robustness of the proposed tracking framework, all experiments were conducted under real industrial welding conditions, including strong arc interference, metal spatter, smoke, and transient illumination variation. No offline replay, manual correction, or post-processing operations were applied to the acquired image sequences, ensuring that the experimental results reflect practical operating conditions in automated welding systems.

### 3.1. YOLO Model Training

**Figure 3:**
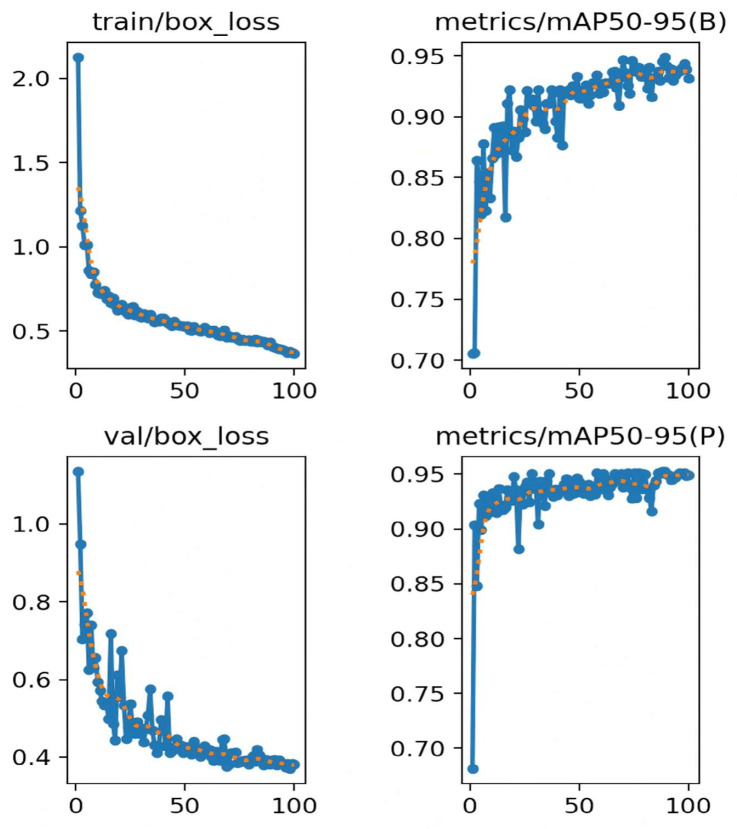
Training and validation performance curves of the YOLO. Note: The bounding box regression losses (**left**) show a monotonic exponential decay for both training and validation sets, while the mean Average Precision metrics mAP50-95 (**right**) converge stably above 93%, establishing a high-fidelity baseline for the subsequent fusion layer.

To establish the semantic measurement module for weld seam localization, the YOLOv8 detector was trained using a dataset containing 1016 manually labeled welding images captured under relatively stable welding conditions with limited arc interference. The training and validation performances over 100 epochs are shown in Figure 3.

As illustrated in Figure 3, both the training loss and validation loss decrease steadily during the training process and gradually converge after approximately 60 epochs, indicating stable network optimization and good convergence behavior. Meanwhile, the detection accuracy metrics continuously improve with increasing training epochs. The final mean Average Precision (mAP@0.5:0.95) reaches 93.5% for bounding box detection and 94.8% for weld feature localization, demonstrating that the trained model can accurately extract weld seam features under normal welding conditions.

Although the detector achieves satisfactory localization accuracy in stable environments, its performance may degrade under severe arc interference, illumination saturation, and temporary feature loss. Therefore, the trained YOLO detector is further integrated with the proposed confidence-guided fusion framework to improve tracking robustness and continuity under complex industrial welding conditions.

### 3.2. Hyperparameter Selection and Analysis

**Figure 4:**
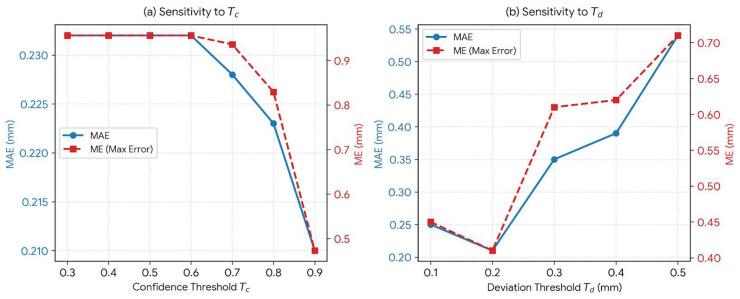
Sensitivity analysis of tracking performance (MAE and ME) with respect to confidence threshold $T_{c}$ and spatial deviation threshold $T_{d}$ across test sequences.

To systematically determine the optimal decision parameters without loss of generality, the parameter sensitivity analysis was conducted on the evaluation dataset. The confidence threshold $T_{c}$ and spatial deviation threshold $T_{d}$ were swept across their functional operating ranges to observe their combined effect on tracking accuracy (MAE) and maximum deviation (ME). The results are illustrated in Figure 4.

Hyperparameter analysis over representative test sequences reveals distinct error-response behaviors for $T_{c}$ and $T_{d}$: elevating $T_{c}$ from 0.3 to 0.9 monotonically suppresses errors—saturating below 0.6 but halving the ME from 0.956 mm to 0.473 mm at $T_{c}\geq 0.8$ by filtering low-quality visual features—while $T_{d}$ exhibits a convex sensitivity profile, where $T_{d}$ = 0.2 mm achieves the optimal trade-off (MAE = 0.21 mm, ME = 0.41 mm) by balancing over-filtering prediction drift ($T_{d}$ = 0.1 mm) and spatter-induced noise admission ($T_{d}\geq 0.3$ mm). Consequently, the fixed baseline configuration ($T_{c}=0.8$, $T_{d}$ = 0.2 mm) was frozen and applied across all subsequent evaluation scenarios.

### 3.3. Tracking Performance and Robustness Analysis

**Figure 5:**
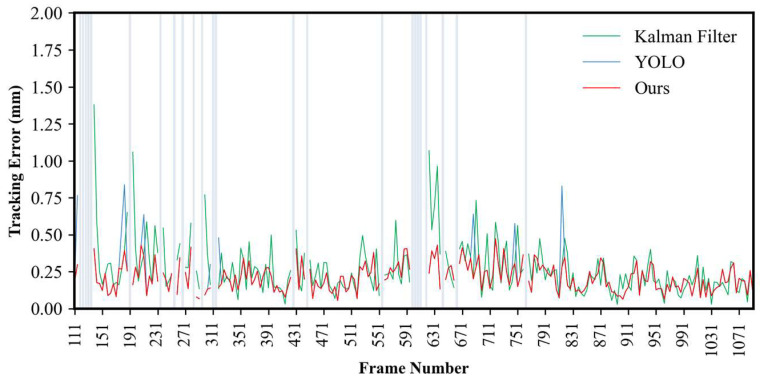
Dynamic tracking error trajectories of the compared methods on industrial weld seam image sequences. Light gray shaded regions denote intervals of severe arc-induced visual degradation, during which weld seam features become unobservable and reliable manual annotation is unavailable. Tracking trajectories are therefore truncated within these intervals. Benefiting from scene-aware reliability estimation and adaptive measurement regulation, the proposed SACG-YOLO framework maintains a consistently bounded tracking error and rapidly restores accurate localization once reliable visual observations become available.

To comprehensively evaluate the tracking accuracy and dynamic robustness of the proposed framework, a continuous 1091-frame industrial welding experiment is conducted under non-stationary arc interference conditions. Figure 5 presents the temporal evolution of tracking errors for the three compared methods across the entire sequence. For quantitative evaluation, the sequence is further partitioned into three representative operating scenarios according to interference severity: nominal conditions, weak arc interference, and severe arc interference. The corresponding statistical indicators, including mean error, maximum error, and standard deviation, are summarized in Table 1.

From a global perspective, the results in Figure 5 reveal increasingly divergent performance trends among the three methods as interference intensity increases, which is further corroborated by the quantitative results in Table 1. The standalone YOLO detector exhibits unstable behavior under dynamic interference. Although it maintains acceptable accuracy under nominal conditions, it suffers from intermittent tracking failures under strong arc disturbances, resulting in missing or invalid outputs in the temporal sequence. This indicates limited robustness to non-stationary noise and abrupt illumination changes.

In contrast, the conventional Kalman filter maintains temporal continuity even under detection failures, demonstrating its advantage in state prediction during missing observations. However, its performance is characterized by significant error fluctuations under interference, with pronounced peak deviations during severe arc disturbance periods. This is reflected in Table 1 by a relatively high standard deviation and a maximum error reaching 1.379 mm, indicating limited adaptability to non-Gaussian and time-varying noise conditions.

In comparison, the proposed SACG-YOLO consistently achieves the most stable performance across all scenarios. Benefiting from the confidence-guided switching mechanism between detection and prediction, the proposed method maintains a low-error and low-variance trajectory throughout the sequence. Under nominal conditions, it achieves the lowest mean error of 0.155 mm with a standard deviation of 0.054 mm, demonstrating high steady-state accuracy. Under weak interference, it effectively suppresses transient error peaks, achieving a 48.8% reduction in maximum error compared with the standalone YOLO detector. More importantly, under severe arc interference, the proposed method avoids catastrophic divergence and constrains residual tracking error within 0.406 mm, indicating strong robustness under extreme non-stationary conditions. The performance improvement is not merely attributed to temporal filtering. Instead, it primarily stems from the proposed scene-aware reliability estimation mechanism, which explicitly evaluates the degradation level of the current welding scene and prevents unreliable YOLO observations from contaminating the temporal state estimation process. As a result, the proposed framework achieves both improved localization accuracy and enhanced tracking continuity under dynamically varying arc interference.

Overall, these results demonstrate that the proposed framework provides a superior balance between accuracy and robustness across varying interference levels. The following subsections further analyze the performance differences by examining the internal switching mechanism and scenario-specific behavior in detail.

#### 3.3.1. Scenario I: Tracking Accuracy Under Stable Conditions

**Figure 6:**
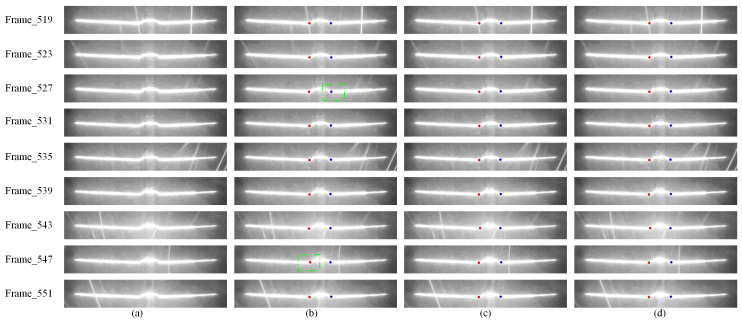
Qualitative comparison of weld seam tracking and dynamic ROI localization under stable welding conditions. (**a**) Original image sequence showing a clearly observable laser stripe and weld groove. (**b**) Tracking result of the conventional Kalman filter. (**c**) Tracking result of the standalone YOLO detector. (**d**) Tracking result of the proposed SACG-YOLO framework. The frame index shown on the left identifies the corresponding image in the welding sequence. Red and blue markers denote the automatically localized left and right weld seam feature points (groove boundaries), respectively, while green dashed boxes highlight regions exhibiting comparatively larger localization deviations.

Under stable welding conditions, the arc plasma remains stable, and localized illumination disturbances are minimal, allowing all three evaluated methods to operate under relatively ideal imaging conditions. Figure 6 provides a comparative visualization of feature point tracking results for representative frames extracted from a steady-state welding sequence. The first column (Figure 6a) presents the original image sequence captured during hollow spherical welding, where the laser stripe exhibits stable intensity distribution and well-defined groove boundaries. The annotated frame indices (e.g., Frames 519–551) correspond to representative samples selected from the stable operating stage. In all subfigures, red and blue markers denote the left and right weld seam feature points, respectively, while green dashed boxes highlight regions with comparatively larger localization deviations.

Under this stable condition, all three methods achieve continuous and structurally consistent seam tracking without catastrophic failure. However, differences in temporal stability and spatial responsiveness are still observable. As shown in Figure 6b, the traditional Kalman filter exhibits noticeable temporal lag in certain frames (e.g., frames 527 and 547), where estimated feature positions deviate from the true laser stripe centerline. This behavior is primarily attributed to the model mismatch between the constant-velocity motion assumption and the slight nonlinear variations in weld seam geometry, as well as sensitivity to localized intensity fluctuations. These factors lead to cumulative prediction delay and result in a maximum tracking error of 0.573 mm.

Figure 6c presents the results of the standalone YOLO detector. By performing frame-wise feature inference, YOLO effectively eliminates temporal accumulation error and achieves improved spatial alignment with the weld seam structure. However, minor inter-frame variations are still observed, primarily caused by local arc reflections and subtle illumination perturbations, which introduce small fluctuations in feature localization.

In contrast, the proposed SACG-YOLO framework shown in Figure 6d achieves the most stable tracking performance across all sampled frames. The detected feature points remain consistently aligned with the weld seam centerline, and the ROI updates exhibit strong temporal consistency with reduced jitter. Under nominal welding conditions, the estimated scene confidence remains consistently high, indicating that the weld seam is clearly observable and only minimally affected by arc-induced degradation. Consequently, measurement regulation rarely suppresses valid detections, allowing reliable YOLO observations to be fully incorporated into state estimation while simultaneously improving temporal consistency through Kalman-based prediction. As a result, the proposed method achieves the lowest steady-state mean tracking error of 0.155 mm while maintaining highly consistent localization performance across the entire sequence.

Overall, although all three methods are capable of stable seam tracking under nominal welding conditions, their robustness characteristics differ significantly. The Kalman filter is limited by model assumptions that induce temporal lag, whereas YOLO improves spatial responsiveness but remains sensitive to local visual disturbances. By integrating adaptive scene-aware decision-making with temporal state estimation, the proposed framework achieves improved tracking stability and higher localization accuracy under nominal operating conditions.

#### 3.3.2. Scenario II: Tracking Stability Under Weak Arc Interference

**Figure 7:**
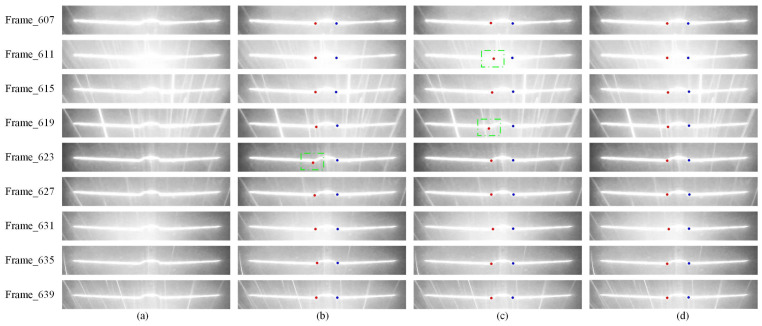
Qualitative comparison of weld seam tracking and dynamic ROI localization under weak arc interference. (**a**) Original image showing localized arc-induced visual degradation, including plasma flickering and localized overexposure. (**b**) Tracking result of the conventional Kalman filter. (**c**) Tracking result of the standalone YOLO detector. (**d**) Tracking result of the proposed SACG-YOLO framework. The frame index shown on the left identifies the corresponding image in the welding sequence. Red and blue markers denote the automatically localized left and right weld seam feature points (groove boundaries), respectively, while green dashed boxes highlight regions exhibiting comparatively larger localization deviations.

When the welding process is subject to weak arc interference, the imaging environment is characterized by plasma flickering, localized overexposure, and intermittent spatter-induced reflections. These factors jointly degrade the radiometric consistency and structural continuity of the laser stripe. Figure 7 presents a comparative visualization of tracking results under such non-stationary disturbances. Compared with nominal conditions, the image sequence exhibits significant intensity fluctuations and localized arc-induced streaks, particularly in the regions around Frames 611–619 and 623–639, where radiometric instability becomes more pronounced.

Under these conditions, performance differences among the three tracking paradigms become increasingly evident. As shown in Figure 7b, the Kalman filter exhibits noticeable temporal drift and accumulated estimation bias. The highlighted regions indicate progressive deviations between predicted feature locations and the true weld seam centerline. This degradation is primarily attributed to the mismatch between the constant-velocity motion assumption and the actual nonlinear motion of the welding seam under arc-induced perturbations, as well as increased uncertainty in the observation process caused by radiometric noise. As a result, prediction errors accumulate over time, leading to a maximum tracking error of 0.742 mm and a standard deviation of 0.129 mm, reflecting limited robustness under time-varying disturbances.

Figure 7c illustrates the performance of the standalone YOLO detector. By performing frame-wise inference, YOLO eliminates temporal accumulation effects and maintains overall spatial alignment with the weld seam. However, under intensified arc interference, its localization variance increases due to reduced detection confidence in regions affected by overexposure and stripe distortion. This is particularly evident in Frames 611 and 619, where local radiometric disturbances induce noticeable deviations in detected feature positions. Consequently, the maximum tracking error increases to 0.829 mm, while the standard deviation reaches 0.106 mm, indicating reduced stability under weak but non-negligible environmental perturbations.

In contrast, the proposed SACG-YOLO framework shown in Figure 7d maintains stable and consistent tracking performance throughout the sequence. Even under localized glare and intermittent arc reflections, the detected seam features remain closely aligned with the groove centerline, exhibiting limited temporal variation. Although YOLO still produces valid detections under weak arc interference, the estimated scene confidence decreases in localized overexposed regions, indicating partial degradation of weld seam observability. The reduced scene confidence enables the proposed framework to identify localized degradation before substantial localization errors occur, allowing unreliable observations to be selectively suppressed while preserving reliable measurements in unaffected regions. Consequently, transient detection errors are prevented from propagating into the temporal state estimation process, resulting in improved tracking stability under weak arc interference. Quantitatively, the proposed method constrains the maximum tracking error within 0.424 mm, representing a 48.8% reduction compared with the standalone YOLO detector, while also achieving the lowest standard deviation of 0.068 mm.

Overall, under weak arc interference conditions, the Kalman filter is primarily affected by model mismatch and error accumulation, YOLO is limited by sensitivity to radiometric perturbations, whereas the proposed framework demonstrates superior robustness by effectively integrating temporal estimation and adaptive confidence-aware decision-making, resulting in improved stability and reduced variance in tracking performance.

#### 3.3.3. Scenario III: Tracking Robustness Under Severe Arc Interference

**Figure 8:**
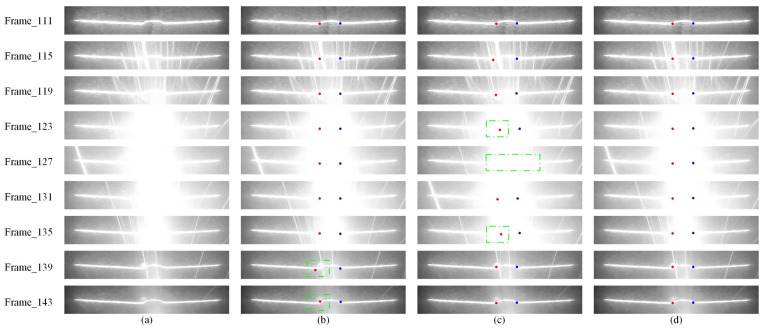
Qualitative comparison of weld seam tracking and dynamic ROI localization under severe arc interference. (**a**) Original image showing severe arc-induced visual degradation characterized by radiometric saturation, plasma blooming, and substantial loss of weld seam visibility. (**b**) Tracking result of the conventional Kalman filter. (**c**) Tracking result of the standalone YOLO detector. (**d**) Tracking result of the proposed SACG-YOLO framework, which maintains continuous localization through scene-aware reliability-guided measurement regulation. The frame index shown on the left identifies the corresponding image in the welding sequence. Red and blue markers denote the automatically localized left and right weld seam feature points (groove boundaries), respectively, while green dashed boxes highlight regions exhibiting comparatively larger localization deviations.

In scenarios with severe arc interference, the visual sensing system operates under an extreme radiometric degradation regime characterized by large-area overexposure, strong plasma blooming, and significant loss of structural visibility. As shown in Figure 8a, the arc intensity progressively increases from Frames 119–131, leading to substantial saturation in the imaging region. During this stage, the laser stripe and groove boundary features are heavily degraded, resulting in a near-complete loss of reliable geometric and semantic information. This condition represents a critical challenge for real-time weld seam tracking, where both measurement observability and feature continuity are severely compromised.

Figure 8b shows the results of the traditional Kalman filter under the same scenario. Unlike YOLO, the Kalman-based approach maintains continuous state propagation throughout the interference period. However, this continuity leads to a gradual increase in state contamination. In the absence of reliable observational data, the filter continues to propagate biased motion estimates, resulting in significant drift accumulation. Even after the visual features reappear, the filter exhibits delayed correction and prolonged misalignment with the true groove centerline, as observed in Frames 139 and 143. This behavior reflects the limitation of model-based temporal propagation under non-Gaussian observation degradation. The maximum tracking error reaches 1.379 mm, indicating poor robustness in extreme radiometric conditions.

Figure 8c illustrates the performance of the standalone YOLO detector under this extreme condition. During the peak interference interval, the detector fails to produce valid feature outputs due to severe overexposure and structural feature disappearance, resulting in tracking failure and invalid estimates. However, an important property can be observed after the disturbance subsides (Frames 135–143): once the weld structure re-emerges, YOLO rapidly restores accurate localization without relying on historical temporal information. This behavior is attributed to its frame-wise independent inference mechanism, which enables fast spatial re-detection but lacks temporal consistency during complete occlusion.

In contrast, the proposed SACG-YOLO framework, shown in Figure 8d, maintains stable tracking throughout the entire interference sequence. During severe radiometric saturation, the scene-aware confidence decreases rapidly, indicating that the current observations are no longer reliable, even when the detector still reports relatively high confidence. Consequently, unreliable measurements are rejected before entering the Kalman update process, preventing state contamination while allowing the tracker to maintain stable prediction-based propagation until reliable observations become available again.

To further investigate long-term error accumulation during visual occlusion, an additional analysis was conducted for Frames 115–139, where severe arc interference continuously degraded weld seam visibility. During this interval, the standalone Kalman filter relied solely on constant-velocity prediction because reliable visual observations were unavailable. Without measurement correction, prediction errors accumulated progressively, increasing the tracking error from 0.298 mm at Frame 115 to 1.379 mm at Frame 139. This result clearly illustrates the inevitable propagation of kinematic errors during prolonged visual occlusion. This observation confirms that the principal limitation of conventional Kalman filtering under prolonged visual occlusion is not the prediction model itself but the absence of reliable measurement updates required to restore estimator accuracy.

By contrast, SACG-YOLO effectively limits this error propagation through adaptive reliability-aware measurement regulation. Once scene observability recovers (Frame 139), reliable YOLO measurements are immediately reintroduced to correct the accumulated prediction error. As a result, the tracking error increases only marginally, from 0.298 mm to 0.406 mm, demonstrating rapid recovery and stable localization after severe interference.

It should be noted that no motion-model-based estimator can completely eliminate prediction error during extremely long visual occlusions because estimation uncertainty inevitably increases without corrective observations. The proposed framework therefore aims to bound, rather than eliminate, error accumulation by suppressing unreliable measurements and rapidly restoring measurement updates when scene observability recovers. For safety-critical industrial applications, a configurable maximum prediction duration can additionally be employed to trigger system warnings or safety intervention when visual observations are unavailable for an extended period, thereby preventing excessive drift.

### 3.4. Reliability Analysis and Ablation Study of the Confidence-Guided Fusion Strategy

To further validate the necessity of the proposed scene-aware confidence-guided fusion strategy, a dedicated ablation study is conducted from both system-level and mechanism-level perspectives. First, the temporal tracking stability of the proposed SACG-YOLO framework is compared with that of a conventional confidence-gated fusion strategy that directly relies on YOLO detector confidence for measurement updates. Subsequently, the intrinsic relationship between detector confidence and spatial localization reliability under welding interference is further analyzed. Through these two complementary evaluations, the proposed experiments aim to demonstrate not only the superiority of the scene-aware fusion mechanism in maintaining stable tracking under severe disturbances but also the fundamental limitation of using detector confidence alone as an indicator of observation reliability in complex industrial welding environments.

#### 3.4.1. Tracking Stability Comparison Between Confidence-Gated and Scene-Aware Fusion Strategies

**Figure 9:**
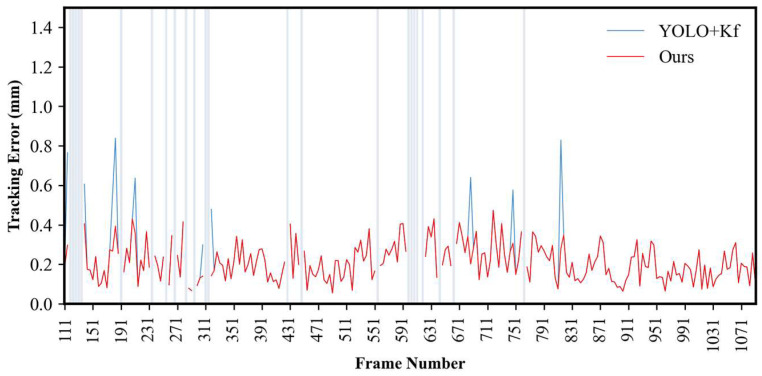
Comparison of temporal tracking error trajectories between the conventional YOLO-confidence-guided strategy and the proposed scene-aware confidence-guided fusion strategy.

To evaluate the effectiveness of the proposed scene-aware confidence guidance strategy, ablation analysis was performed based on the time tracking error trajectory shown in Figure 9. The comparison focuses on the performance of the traditional cascaded tracking framework (YOLO+KF) and the proposed SACG-YOLO under non-stationary welding interference conditions. In the YOLO+KF configuration, the confidence level output by YOLO is directly used for measurement updates and state synchronization without additional scene-aware reliability verification.

As shown in the error trajectories over the 1091-frame sequence, both methods achieve comparable tracking accuracy under stable welding conditions. During relatively interference-free intervals (e.g., Frames 200–800), the tracking errors of both frameworks remain consistently below 0.5 mm, indicating that YOLOv8 provides sufficiently accurate spatial observations when the laser stripe structure remains clear and stable.

However, significant performance differences emerge during transient disturbance periods. Around Frame 115, corresponding to the onset of severe arc ignition, the conventional YOLO+KF framework exhibits a sharp error increase, with the tracking deviation reaching 0.765 mm. This behavior is primarily caused by unreliable high-confidence observations under localized arc-induced overexposure. Although the detector output becomes spatially distorted due to radiometric interference, the semantic confidence score remains sufficiently high to trigger direct measurement updates. Consequently, corrupted observations are incorporated into the Kalman estimation process, leading to state contamination before the tracking system transitions into a prediction-dominant mode.

The influence of this contaminated state propagation becomes more evident during the subsequent severe radiometric saturation interval (Frames 119–135). During this stage, the laser stripe structure becomes heavily degraded, resulting in temporary loss of reliable visual observations. After the seam features gradually reappear near Frame 139, the conventional YOLO+KF framework still exhibits significant recovery hysteresis, with the residual tracking error remaining at 0.606 mm. This delayed convergence indicates that corrupted internal motion states continue influencing the estimation process even after valid observations are restored. A similar instability can also be observed near Frame 915, where the baseline framework produces a large transient error spike of 1.183 mm under localized interference.

In contrast, the proposed SACG-YOLO framework demonstrates substantially improved robustness throughout the entire sequence. By introducing a scene-aware confidence evaluation mechanism based on structural and photometric consistency cues, the proposed method effectively suppresses unreliable observations before they contaminate the temporal estimation process. As indicated by the red trajectory in Figure 9, the proposed framework significantly reduces the transient disturbance response at Frame 115, constraining the peak tracking error to 0.298 mm.

More importantly, because unreliable observations are isolated prior to severe radiometric saturation, the internal motion states remain stable during the degradation interval. Consequently, once valid seam features reappear, the proposed framework rapidly restores accurate localization without the prolonged convergence behavior observed in the conventional YOLO+KF framework. The residual tracking error after feature recovery remains bounded within 0.406 mm at Frame 139.

Overall, the ablation results demonstrate that explicitly modeling scene degradation provides a more reliable criterion for measurement regulation than relying on detector confidence alone. By preventing unreliable observations from entering the Kalman update process, the proposed framework substantially improves disturbance resilience, temporal stability, and post-interference recovery capability.

#### 3.4.2. Evidence of Confidence–Reliability Decoupling Under Severe Arc Interference

**Figure 10:**
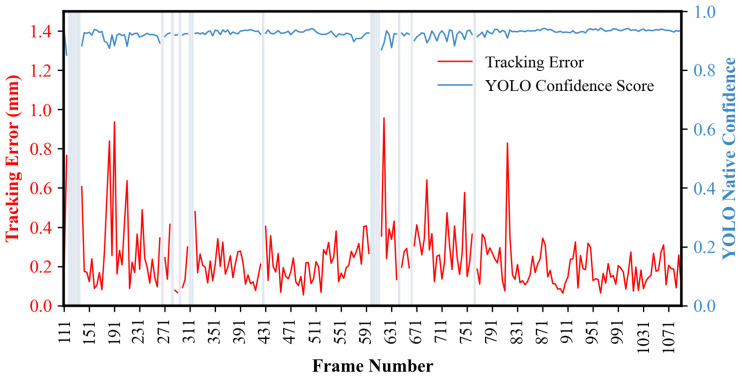
Evidence of confidence–reliability decoupling under severe arc-induced visual degradation. Temporal comparison between YOLO detector confidence and spatial localization error demonstrates that detector confidence no longer reliably reflects localization quality under severe arc interference.

To further investigate the reliability of detector confidence under severe welding interference, the temporal relationship between the YOLO confidence score and the corresponding spatial localization error is analyzed in Figure 10. Unlike conventional confidence-gated tracking paradigms that directly treat detector confidence as a reliable indicator of observation quality, the proposed analysis reveals that high semantic confidence does not necessarily imply accurate spatial localization under complex welding environments.

As shown in Figure 10, the YOLO confidence score remains consistently high throughout most of the tracking sequence, generally fluctuating within the range of 0.90–0.95. Under nominal welding conditions, this high-confidence behavior corresponds well with relatively small tracking errors, indicating that the detector can provide reliable semantic observations when the laser stripe structure remains clear and stable. However, several significant tracking error spikes can still be observed under arc-interference intervals, particularly around Frames 190, 620, and 810, despite the detector maintaining similarly high confidence levels.

This phenomenon reveals a clear decoupling between detector confidence and spatial localization reliability under radiometric degradation conditions. Specifically, severe arc interference introduces local overexposure, plasma blooming, and stripe deformation, which may preserve strong semantic activation within the neural detector while simultaneously corrupting the geometric accuracy of weld seam localization. Consequently, although the detector continues to assign high confidence scores to the predicted weld seam region, the extracted feature positions may exhibit substantial spatial deviation from the true groove boundaries.

The observed confidence-reliability inconsistency further explains the instability of conventional confidence-gated fusion strategies. When detector confidence alone is directly used as the measurement acceptance criterion, corrupted observations generated during strong interference phases may still be incorporated into the temporal estimation process, leading to state contamination, trajectory overshoot, and post-disturbance recovery hysteresis. Therefore, detector confidence alone cannot reliably characterize observation quality in highly dynamic welding environments.

In contrast, the proposed SACG-YOLO framework introduces an additional scene-aware reliability evaluation mechanism that jointly considers structural consistency and photometric variation within the dynamically updated ROI. Instead of relying solely on detector confidence, the proposed method explicitly estimates the physical reliability of visual observations before state correction. This reliability-aware regulation mechanism effectively suppresses semantically overconfident but spatially unreliable measurements under severe arc interference, thereby improving temporal stability and maintaining robust weld seam localization throughout dynamically changing industrial welding conditions.

Overall, the results in Figure 10 demonstrate that semantic confidence and spatial localization reliability may become significantly inconsistent under severe radiometric interference. This observation not only validates the necessity of the proposed scene-aware confidence-guided fusion strategy but also demonstrates that explicit scene degradation assessment is essential for reliability-aware weld seam tracking under severe arc-induced visual degradation.

### 3.5. Runtime Performance and Computational Efficiency

To evaluate the real-time deployment feasibility and computational efficiency of the proposed framework in edge industrial environments, a detailed latency breakdown was benchmarked on a standard CPU-only computing platform without GPU acceleration, as summarized in Table 2.

As reported in Table 2, the primary computational bottleneck stems from the neural network inference (YOLOv8 Detection), which consumes 12.11 ms per frame (95.23% of the total execution time). In contrast, the proposed Scene-Aware Confidence Guidance combined with the linear Kalman filter state update requires an exceptionally lightweight execution time of only 1.10 ms (4.77% of the overall pipeline). The end-to-end processing latency for the entire pipeline is 13.21 ms per frame, corresponding to an inference throughput of ~75.70 FPS. This speed substantially exceeds the standard industrial camera acquisition rate (30 FPS), demonstrating that the SACG mechanism introduces negligible computational overhead while imparting strong disturbance rejection capability, confirming its suitability for real-time industrial deployment.

## 4. Discussion

### 4.1. Reliability-Aware State Estimation Under Dynamic Observability Conditions

The experimental results indicate that the primary challenge of weld seam tracking under severe arc interference is not merely reduced detection accuracy but the degradation of measurement reliability. Under stable welding conditions, detector confidence generally correlates well with localization accuracy because the weld seam remains clearly observable. However, this relationship progressively weakens as arc interference intensifies. Radiometric saturation, plasma blooming, and local feature degradation may substantially distort the geometric structure of the weld seam while the detector still produces high-confidence predictions. Consequently, detector confidence alone is insufficient to characterize the reliability of visual observations under severe welding interference.

This inconsistency explains the limitations of conventional confidence-guided tracking methods. When detector confidence is directly used for measurement updating, unreliable observations may still be incorporated into the Kalman filter, resulting in state contamination, drift accumulation, and delayed recovery after visual degradation. Conversely, relying exclusively on motion prediction may gradually increase estimation errors during prolonged occlusions because the assumed constant-velocity model cannot fully capture complex welding dynamics.

The proposed SACG-YOLO framework addresses this problem by introducing scene-aware confidence as an additional reliability constraint. Instead of relying solely on semantic confidence, the proposed framework jointly evaluates structural consistency and photometric variation within the local welding region to estimate scene observability. The resulting reliability estimate is then used to adaptively regulate the Kalman filter update process. Reliable measurements are incorporated into state estimation, whereas unreliable observations are suppressed until the scene quality recovers.

This adaptive estimation strategy provides two important advantages. First, it effectively prevents unreliable measurements from contaminating the filter state during severe arc interference, thereby reducing tracking instability and error propagation. Second, once the weld seam becomes observable again, reliable measurements are immediately reintroduced, enabling rapid localization recovery without significant temporal drift. These characteristics explain the superior robustness and localization continuity observed in the experimental results.

Overall, the experimental results suggest that robust weld seam tracking should be formulated as a reliability-aware state estimation problem rather than a conventional confidence-thresholding problem. Explicitly modeling scene reliability enables a more effective balance between visual observations and temporal prediction, resulting in improved tracking accuracy, robustness, and continuity under dynamically changing welding conditions.

### 4.2. Robustness Under Severe Arc Interference and Saturation-Dominated Degradation

Severe arc interference represents one of the most challenging scenarios for industrial weld seam tracking because intense plasma radiation produces radiometric saturation, localized overexposure, and feature washout, substantially degrading the observability of weld seam structures. As illustrated in Figure 7, the interference intensity increases significantly during Frames 119–131, where large portions of the laser stripe and groove boundaries become partially or completely obscured. Under these conditions, robust tracking depends not only on detector accuracy but also on the ability to distinguish reliable from unreliable visual observations.

The experimental results reveal two fundamentally different failure mechanisms for conventional tracking approaches. Standalone YOLO exhibits measurement collapse during severe saturation. Once discriminative weld seam features are overwhelmed by arc radiation, localization accuracy deteriorates rapidly and tracking interruptions occur. Although the detector can recover quickly after the interference weakens because each frame is processed independently, the experiments demonstrate that detector confidence may remain relatively high despite significant localization errors. This observation confirms that detector confidence alone is insufficient for assessing measurement reliability under severe radiometric degradation.

In contrast, the Kalman filter maintains continuous state propagation during temporary observation loss but suffers from progressive error accumulation. Corrupted measurements introduced before or during severe interference gradually contaminate the internal state estimate, and the accumulated bias continues to propagate throughout the occlusion period. Consequently, even after reliable observations become available again, the filter requires a relatively long convergence interval before accurate tracking can be restored. This delayed recovery explains the persistent overshoot observed in the experimental results.

The proposed SACG-YOLO framework overcomes these limitations by explicitly incorporating scene-aware confidence into the estimation process. Instead of relying solely on detector confidence, the proposed framework evaluates scene observability through structural consistency and photometric variation, allowing unreliable observations to be identified before they affect state estimation. During severe arc interference, degraded measurements are suppressed, and the tracker temporarily relies on motion prediction using previously validated states. Once scene quality recovers, reliable observations are immediately reintroduced, enabling rapid and stable tracking recovery without significant state drift.

These mechanisms are consistent with the quantitative results. Compared with standalone YOLO, which suffers from tracking interruption, and the conventional Kalman filter, which exhibits substantial drift accumulation, SACG-YOLO maintains continuous weld seam localization throughout severe interference. The maximum tracking error remains limited to 0.406 mm, demonstrating that explicit measurement reliability assessment substantially improves robustness under highly non-stationary welding conditions.

Overall, the experimental results indicate that the principal challenge under severe arc interference is not simply improving detector accuracy but determining whether the current visual observation is trustworthy. Explicitly incorporating scene-aware reliability into the state estimation process enables a more effective balance between visual observations and temporal prediction, thereby significantly improving tracking continuity and robustness in practical industrial welding environments.

### 4.3. Practical Implications, Limitations, and Future Extensions

The proposed SACG-YOLO framework demonstrates potential for practical deployment in industrial robotic welding systems. By providing continuous and reliable weld seam localization under severe arc interference, it supplies stable visual measurements for downstream trajectory correction and adaptive welding control. Moreover, the scene-aware confidence module introduces only minimal computational overhead because it relies on lightweight structural and photometric descriptors rather than computationally intensive neural networks, thereby preserving real-time performance on resource-constrained industrial platforms.

Although developed for weld seam tracking, the proposed reliability-aware estimation strategy is not limited to robotic welding. Since the framework explicitly evaluates measurement reliability before state updating, it can potentially be extended to other industrial vision applications operating under dynamically changing observation conditions, such as laser-guided manufacturing, additive manufacturing, plasma-assisted processing, and high-temperature inspection.

Several limitations should be acknowledged. First, the adopted constant-velocity Kalman filter assumes locally smooth motion. Under prolonged visual occlusion, abrupt nonlinear seam motion, or severe mechanical vibration, prediction uncertainty inevitably increases because reliable corrective observations are unavailable. Second, the scene-aware confidence is constructed from hand-crafted structural and photometric descriptors. Although these descriptors effectively characterize typical arc-induced degradation, they may not fully capture more complex uncertainty sources encountered in diverse industrial environments. Third, the current experimental validation was conducted on an industrial hollow-sphere welding platform. While this platform represents a particularly challenging scenario with continuously varying seam curvature and severe arc interference, it does not encompass all industrial welding geometries. Since the proposed framework regulates state estimation according to observation reliability rather than workpiece geometry, its underlying methodology is expected to be transferable to other welding scenarios. Nevertheless, additional validation is required to comprehensively assess its generalization capability.

Future work will focus on three directions. First, multimodal sensing, including infrared imaging, arc-voltage signals, acoustic emission, and three-dimensional geometric measurements, will be integrated to improve observation reliability under severe optical degradation. Second, learning-based uncertainty estimation will be investigated to replace hand-crafted scene descriptors while maintaining real-time performance. Finally, more advanced motion models together with validation on diverse weld geometries, including straight seams, curved seams, and different joint configurations, will be explored to further improve robustness and verify the generalization capability of the proposed framework for large-scale industrial deployment.

## 5. Conclusions

This paper proposed a scene-aware confidence-guided YOLO framework for robust weld seam tracking under severe arc interference. The proposed method was motivated by the observation that detector confidence alone cannot reliably represent localization quality under severe radiometric degradation, making conventional confidence-based measurement fusion unreliable in practical welding environments.

To address this limitation, a scene-aware confidence estimation mechanism was developed to jointly evaluate structural consistency and photometric variation, thereby characterizing the physical observability of the weld seam. The estimated scene confidence is combined with detector confidence to assess measurement reliability, enabling adaptive regulation of the Kalman filter update process. Consequently, reliable observations are incorporated into state estimation, while degraded measurements are rejected before contaminating the tracking state.

Experimental results demonstrate that SACG-YOLO consistently outperforms conventional YOLO-based tracking under nominal, weak, and severe arc interference conditions. Compared with standalone YOLO and the conventional Kalman filter, the proposed framework effectively suppresses transient localization fluctuations, reduces drift accumulation, and maintains continuous weld seam tracking during severe radiometric saturation. Even under the most challenging interference conditions, the maximum tracking error is constrained to 0.406 mm, demonstrating superior robustness and tracking continuity.

More importantly, this work suggests that robust visual tracking in degraded industrial environments should be formulated as a measurement reliability-aware state estimation problem, rather than relying solely on detector confidence for measurement fusion. By explicitly evaluating scene observability before state correction, the proposed framework provides a lightweight and computationally efficient solution without requiring additional sensing hardware or computationally intensive auxiliary neural networks. Although developed for robotic welding, the proposed reliability-aware estimation strategy is expected to be applicable to a wider range of industrial vision tasks operating under dynamically varying observation conditions.

Future work will focus on integrating multimodal sensing, learning-based uncertainty estimation, and more advanced motion models to further improve robustness under complex industrial environments. In addition, the proposed framework will be validated on more diverse welding geometries, including straight seams, curved seams, and different joint configurations, to comprehensively evaluate its generalization capability across practical industrial welding applications.

## Figures and Tables

**Table 1:** Quantitative tracking error performance under different operational scenarios (mm).

Scenario	Method	MAE	ME	SD
Stable Conditions	YOLO	0.171	0.395	0.063
	Kalman Filter	0.198	0.573	0.088
	Ours	0.155	0.354	0.054
Weak Arc Interference	YOLO	0.492	0.829	0.106
	Kalman Filter	0.428	0.742	0.129
	Ours	0.306	0.424	0.068
Severe Arc Interference	YOLO	0.287	0.765	0.195
	Kalman Filter	0.543	1.379	0.372
	Ours	0.252	0.406	0.094

**Table 2:** Runtime Performance and Computational Cost Breakdown.

Execution Stage	Latency per Frame (ms)	Time Proportion (%)
YOLOv8 Detection	12.11	95.23
SACG & KF State Update	1.10	4.77
Full Pipeline (Total)	13.21	100

## Data Availability

The original contributions presented in this study are included in the article. Further inquiries can be directed to the corresponding author.

## Abbreviations

The following abbreviations are used in this manuscript:

KF	Kalman Filter
ROI	Region of interest
MAE	Mean Absolute Error
ME	Maximum Error
SD	Standard Deviation
YOLO	You Only Look Once
SACG-YOLO	Scene-Aware Confidence-Guided YOLO
